# FLU-v, a Broad-Spectrum Peptide-Based Influenza Vaccine, Induces NK Cell Activating IgG1 and IgG3 Subclass Antibodies in Humans

**DOI:** 10.3390/vaccines13111084

**Published:** 2025-10-22

**Authors:** Lisbeth M. Næss, Hillary Vanderven, Diane Bryant-Bratlie, Ida Laake, Olga Pleguezuelos, Fredrik Oftung

**Affiliations:** 1 Department of Infection Control and Vaccines, Division of Infection Control, Norwegian Institute of Public Health (NIPH), P.O. Box 222, Skøyen, N-0213 Oslo, Norway; 2 Biomedical Sciences and Molecular Biology, College of and Australian Institute of Tropical Health and Medicine, James Cook University (JCU), Douglas, QLD 4811, Australia; hillary.vanderven@jcu.edu.au; 3 Department of Microbiology and Immunology, Peter Doherty Institute, University of Melbourne, Melbourne, VIC 3000, Australia; 4 Department of Methods Development and Analytics, Division of Infection Control, Norwegian Institute of Public Health (NIPH), P.O. Box 222, Skøyen, N-0213 Oslo, Norway; dlb.bratlie@gmail.com (D.B.-B.); ida.laake@fhi.no (I.L.); fredrik.oftung@fhi.no (F.O.); 5 ConserV Bioscience Ltd., Heyford Park Innovation Centre, 77 Heyford Park, Bicester OX25 5HD, UK; olga.pleguezuelos@conservbio.com

**Keywords:** universal influenza vaccine, FLU-v, clinical trial, IgG subclass, NK cell activation, ADCC

## Abstract

**Background/Objectives:** FLU-v is a peptide-based broad-spectrum influenza vaccine proven to induce humoral and cellular immune responses in humans. In this study, FLU-v-specific IgG1 and IgG3 subclass antibodies, induced by adjuvanted or non-adjuvanted FLU-v vaccination in healthy adults participating in a phase II clinical study, were quantitated. The ability of these antibodies to induce NK cell activation was investigated. **Methods:** An ELISA was developed to quantify FLU-v-specific IgG1 and IgG3 antibodies in serum. A flowcytometric assay based on an NK cell line was used to evaluate NK cell activation by expression of degranulation marker CD107a. **Results:** In the adjuvanted FLU-v group, IgG1 and IgG3 seroconversion on day 42 was 88.5% and 86.5% compared to 53.4% and 29.3% in the non-adjuvanted FLU-v group, which was significantly different from the respective placebo groups (0–6.3%). Adjuvanted FLU-v vaccination induced a raise in median IgG1 and IgG3 levels from 435 and 167 ng/mL pre vaccination to 4422 and 2020 ng/mL 42 days post vaccination, representing a fold increase of 16.3 for IgG1 and 11.6 for IgG3, which was sustained on day 180 post vaccination (10.4-fold and 5.0-fold, respectively). Non-adjuvanted vaccination induced a more modest increase in IgG1 and IgG3 from 655 and 206 ng/mL pre vaccination to 1808 and 264 ng/mL 42 days post vaccination. A correlation between levels of FLU-v-specific IgG, IgG1, or IgG3 and their ability to induce NK cell activation was demonstrated. **Conclusions:** A single dose of adjuvanted FLU-v induced high levels of long-lasting antigen-specific IgG1 and IgG3 antibodies with NK cell-mediated effector functions relevant to protection against influenza disease.

## 1. Introduction

Influenza vaccines that provide broad protection against diverse influenza strains are highly needed to provide sustainable seasonal protection and pandemic preparedness against emergent influenza strains [1]. A promising approach has focused on the use of peptides from conserved regions of various influenza proteins for the induction of protective antibody- and cell-mediated immune responses [2,3].

FLU-v is a broad-spectrum influenza vaccine that consists of four peptides originating from conserved regions of the matrix 1 (M1) and matrix 2 (M2) proteins, nucleoprotein A (NP-A), and nucleoprotein B (NP-B) of the influenza virus [3], synthetically manufactured using F-moc chemistry [4]. FLU-v aims to provide broad protection against influenza A and B strains. FLU-v has been demonstrated to induce humoral and cross-reactive cellular immune responses in both preclinical [3] and clinical studies [5,6,7,8] and to provide protection against mild-to-moderate influenza disease in a H1N1 human influenza challenge study [9]. In a phase IIb clinical trial, immune responses after two doses of non-adjuvanted FLU-v or a single dose of adjuvanted FLU-v [10] were compared [7,11] and showed that a single dose of adjuvanted FLU-v induced robust Th1 responses characterized by an increase in IFN-γ-, TNF-α-, and IL-2-producing cells [7]. In addition, FLU-v-induced IFN-γ and granzyme B cellular responses were demonstrated to be cross-reactive against multiple inactivated influenza strains in vitro [8]. Adjuvanted FLU-v vaccine also induced strong long-lasting FLU-v-specific IgG antibodies [7], but due to internal location of the viral antigens, these antibodies have no neutralizing activity.

It has been shown that the majority of IgG antibodies generated after influenza infection belong to the IgG1 subclass, followed by a smaller proportion of IgG3, and negligible amounts of IgG2 and IgG4 [12,13]. IgG1 and IgG3 are also superior to IgG2 and IgG4 in inducing Fc-mediated effector functions relevant to protection against influenza, such as opsonophagocytosis and antibody-dependent cellular cytotoxicity (ADCC) [14]. Thus, the development of broad-spectrum influenza vaccines focusing on the induction of antibodies with Fc receptor (FcR)-mediated effector functions, in addition to neutralization potential, has gained interest [15].

In humans, natural killer (NK) cells are considered the primary effector cell for ADCC of influenza-infected cells. ADCC is initiated by influenza antigen–IgG complexes binding to FcγRIIIa on NK cells via their Fc region, leading to activation of NK cell cytotoxic activity [16]. In the mouse model, it has been demonstrated that broadly cross-reactive antibodies directed against HA, M2, and NP contribute to protection against influenza virus challenge by activating Fc-mediated effector functions [16]. Human anti-M2 and anti-NP IgG antibodies have also been shown to activate NK cells [17,18], and NK cell activating antibodies have recently been proposed as a correlate of protective immunity against influenza infection [19]. Moreover, a small human influenza challenge study showed that subjects with elevated ADCC-inducing antibody titers experienced lower disease severity and reduced viral load [19]. Induction of NK cell activating IgG1 and IgG3 antibodies directed against conserved influenza antigens is therefore a promising strategy towards the development of a broad-spectrum influenza vaccine.

In this study, we have developed and validated an Enzyme-Linked Immunosorbent Assay (ELISA) to quantify FLU-v-specific IgG1 and IgG3 antibodies in human serum and used this assay to measure IgG subclass responses in serum obtained from volunteers in a phase IIb study, who were vaccinated with adjuvanted and non-adjuvanted FLU-v [7]. In addition, we have explored the functional role these non-neutralizing antibodies may play by evaluating their ability to activate NK cell cytotoxicity, which is essential for ADCC of influenza-infected cells in vivo.

## 2. Materials and Methods

### 2.1. Vaccine and Clinical Trial Design

FLU-v is composed of an equimolar mix (50 nmols of each peptide) of four lyophilized synthetic peptides (between 19 and 32 aa in length) originating from conserved regions of the M1, NPA, NPB, and M2 influenza virus proteins, as shown in Table 1 [7].

The immunogenicity of FLU-v was evaluated in a randomized, placebo-controlled, double-blind, single-center clinical trial (EudraCT:2015-001932-38) [6] in healthy adults aged 18–60 years, who were randomized to one of four treatment arms (ratio 2:2:1:1). Subjects in the non-adjuvanted FLU-v group (*n* = 58) received two doses of FLU-v, 21 days apart, as a suspension in 0.25 mL of 0.01M HCl and 0.25 mL of 0.01M NaOH. Subjects in the adjuvanted FLU-v group (*n* = 52) received one dose of FLU-v as a water-in-oil emulsion made with 0.25 mL of Montanide ISA-51 [10] and 0.25 mL of water for injection (WFI), followed by one dose of 0.5 mL of saline 21 days later. Non-adjuvanted placebo subjects (*n* = 32) received two 0.5 mL doses of saline 21 days apart, and adjuvanted placebo subjects (*n* = 26) received one dose of 0.5 mL emulsion prepared with 0.25 mL of WFI and 0.25 mL of Montanide ISA-51, followed by 0.5 mL of saline 21 days later. All injections were given subcutaneously. Blood samples were obtained before vaccination on day 0 and on days 42 and 180 post vaccination. Study approval was obtained by the Dutch Central Committee on Research Involving Human Subjects (reference NL55061.000.15) and the Dutch Ministry of Health, Welfare and Sport.

### 2.2. Quantitation of FLU-v-Peptide-Specific IgG1 and IgG3 Subclass Antibodies

An ELISA protocol was developed to quantify FLU-v-specific IgG1 and IgG3. Test wells of microtiter plates (Maxisorp, Nunc, Thermo Fisher Scientific, Waltham, MA, USA) were coated with 100 µL of FLU-v antigen at 2 μM (0.5 µM of each of the four peptides) in phosphate-buffered saline (PBS). Wells in the standard rows were coated with human IgG1 or IgG3 reference antibodies (16-16-090707-1/16-16-090707-3, Athens Research, Athens, GA, USA), performing double decreasing serial dilutions starting at 1000 ng/mL up to 1.95 ng/mL. PBS was used as a blank. Plates were incubated at +4 °C overnight and washed twice with PBS-Tween. After blocking with PBS-Tween-1% BSA (200 µL/well) for 1 h at room temperature (RT), plates were washed five times with PBS-Tween. Serum samples, diluted 1:50 in PBS-Tween-1% BSA, were added to the test wells (100 µL/well), and PBS-Tween-1% BSA was added to standard wells (100 µL/well). Plates were incubated for 2 h at RT, washed 5 times with PBS-Tween, followed by addition of 100 µL/well of alkaline-phosphatase-conjugated anti-IgG1 (1:1000 dilution, ab9773, Abcam, Cambridge, UK) or anti-IgG3 (1:3000 dilution, 9210-04, Southern Biotech, Birmingham, AL, USA) detection antibodies, and incubated for 1 h at RT. Plates were washed 5 times with PBS-Tween, and 100 µL/well of phosphatase substrate (pNPP, Sigma-Aldrich, St. Louis, MO, USA) were added. Plates were allowed to develop for 35 min, and the reaction was stopped with 50 µL/well of stop solution (3M NaOH). Absorbance was measured at 405 and 450 nm with an absorbance microplate reader (BioTek ELx808, BioTek Instruments, Winooski, VT, USA). Calculations were based on optical density (OD) values obtained at 450 nm (reference wavelength) that were subtracted from OD values at 405 nm (test wavelength). A standard curve was plotted using Gen5, v2.04.11 software (BioTek Instruments, Winooski, VT, USA) and used to interpolate the antibody concentration in serum test samples. Thus, any sample with OD value above the highest concentration of the standard was diluted and re-tested for the OD to fall within the linear range.

### 2.3. Validation of ELISA Protocol

Due to the lack of a reference sample with known amounts of IgG1 and IgG3 FLU-v-specific antibodies, only limited validation could be performed focusing on precision (intra-assay and intermediate) and dilutional linearity. The IgG1 and IgG3 reference standards were tested in two-fold dilutions from 1000 ng/mL to 1.95 ng/mL. Eight replicates for each of the ten dilutions were assayed on three different days. Each plate also included eight replicate values of blank wells (buffer only) to evaluate the precision of blank well measurements. Precision was evaluated as a coefficient of variation (CV): intra-assay precision as variation in absorbance between replicate wells, and inter-assay precision as variation in absorbance between means of runs on the 3 days. The lower limit of detection (LLD) was defined as the mean of blank wells across 3 different runs (8 replicates per plate) + 2 × standard deviation. Dilutional linearity was investigated using a 4-parameter logistic curve fit from the BioTek Manager software (Gen5 v2.04.11), and linear regression was performed with Microsoft Excel for Windows, version 13.0.

### 2.4. Antibody-Mediated NK Cell Activation Assay

The NK cell line GFP-CD16 (176V) NK-92 [20] was used to perform the NK cell activation assays as previously described [18]. The parental cells of GFP-CD16 (176V) NK-92 are NK-92 cells (ATCC, CRL-2407). GFP-CD16 (176V) NK-92 cells have been transduced with a retrovirus to express the high affinity variant of FcγRIIIa (CD16a, allotype V176) in the pBMN-IRES-EGFP vector. On these cells, surface expression of FcγRIIIa correlates with GFP expression. GFP-CD16 (176V) NK-92 cells were kindly provided by Dr. Kerry Campbell from the Institute for Cancer Research in Philadelphia, PA. Activation of NK cells translate into expression of the CD107a degranulation marker. To assess whether anti-FLU-v antibodies can activate NK cells, 600 ng of FLU-v peptides or non-related peptides were bound to 96-well NUNC Maxisorp plates (Thermo Fisher Scientific, Waltham, MA, USA) overnight at +4 °C. Wells were washed with PBS, blocked with 200 µL of 5% BSA 0.1% Tween 20 PBS for 2 h at 37 °C, and incubated with a 1:10 dilution of serum for 2 h at 37 °C. Next, 2 × 10^5^ GFP-CD16 (176V) NK-92 cells were added to each well and incubated for 5 h at 37 °C with 5% CO_2_. In total, 1 mM EDTA was added to the wells to minimize adherence of the cells to the plate, in addition to anti-CD107a APC-Cy7 (clone H4A3; BioLegend, San Diego, CA, USA). Plates were placed in the dark for 30 min. Cells were washed twice with PBS and fixed with 1% formaldehyde, and the number of GFP-CD16 (176V) NK-92 cells expressing CD107a was acquired on a FACSCanto II flow cytometer (BD Biosciences, San Jose, CA, USA). Blank wells without sera and non-related peptide-coated wells incubated with participant sera were used as negative controls. A subset of subjects was selected for this analysis based on fold-change in FLU-v-specific IgG1 and IgG3 from day 0 to day 42 post vaccination. Whereas the median concentrations for IgG1 and IgG3 in the full set of samples (both active groups combined) were 523 ng/mL (IQR 5.0–1574 ng/mL) and 177 ng/mL (131–260 ng/mL) for day 0 and 2520 ng/mL (750–9003 ng/mL) and 772 ng/mL (225–3125 ng/mL) for day 42, the medians in the subset of samples selected for the NK activation analysis were 5.0 ng/mL (IQR 5.0–1038 ng/mL) and 178 ng/mL (137–258 ng/mL) for day 0 and 2478 ng/mL (1369–12,119 ng/mL) and 3102 ng/mL (1034–6059 ng/mL) for day 42. The subset consisted of fifty day 0 (pre-vaccination) and day 42 paired serum samples including placebo and vaccine groups that also had the lowest background of FLU-v-specific antibodies pre-vaccination to remove volunteers with pre-existing antibody responses.

### 2.5. Statistical Analysis

Non-parametric tests were applied due to non-normal data distributions, as revealed by the D’Agostino and Pearson Omnibus test and visual inspection of histograms and QQ-plots. Wilcoxon’s signed-rank test was used to compare concentrations of IgG subclass antibodies over time within each group. The Mann–Whitney U test was used for comparisons between the vaccine groups and their corresponding placebo group, as stated in the statistical analysis plan for the clinical trial. Vaccine responders were defined as participants showing at least a 2-fold increase in antibody concentrations from pre to post vaccination. *p*-values for comparison of the percentage of responders between groups were calculated with the Fisher mid-P test. For the NK cell activation assay, Wilcoxon’s signed-rank test was used for comparison between time points within each group, but a statistical comparison between placebo and the vaccine groups could not be performed due to the small number of samples in the placebo group. Spearman’s rank correlation coefficients between total IgG, IgG1 or IgG3 antibodies and the percentage of activated NK cells (GFP^+^ CD107a^+^ CD16 (V176) NK-92 cells) were calculated. Statistical analyses were performed using Stata SE16.0 and GraphPad Prism, version 8.1.2 software (GraphPad Prism, San Diego, CA, USA).

## 3. Results

### 3.1. Validation of ELISA Method

CV values for intra-assay precision were found to be <8.2% for IgG1 and <5% for IgG3 at the ten reference concentrations tested in three separate runs (Table S1, Supplementary). CV values for blank wells were found to be <5% (except for one of 24 wells, where the CV was 8.2%). Mean OD values for blank wells in the three runs varied between 0.044 and 0.046. For inter-assay (intermediate) precision, CVs varied from 1.2 to 13.4% for IgG1 (Table S2, Supplementary) and from 1.4 to 9.4% for IgG3 (Table S3, Supplementary), depending on the concentration level of IgG1 and IgG3 reference antibodies. For blank wells, intermediate precision was found to be <3% in the three runs. The lower limit of detection (LLD) was found to be <1.95 ng/mL for IgG1 and 1.95 ng/mL for IgG3. In addition, dilutional linearity of the reference curve was investigated, and a four-parameter logistic curve fit was found to be suitable for both the IgG1 and IgG3 references, with r^2^ values > 0.999. Both IgG1 and IgG3 reference curves showed a dilutional linearity of r^2^ > 0.97 in the concentration range 1000–1.95 ng/mL for IgG1 and 500–1.95 ng/mL for IgG3.

### 3.2. IgG1 and IgG3 Subclass Antibody Responses After FLU-v Vaccination

Vaccine responder frequency: In the adjuvanted FLU-v group, the percentage of vaccine responders was 88.5% for IgG1 and 86.5% for IgG3 on day 42, compared to 0% for both subclasses in the adjuvanted placebo group (*p* < 0.0001 for both) (Table 2). Administration of non-adjuvanted FLU-v resulted in 53.4% and 29.3% responders for IgG1 and IgG3, respectively, compared to 6.3% and 0% in the non-adjuvanted placebo group (*p <* 0.0001 and *p =* 0.0002). On day 180, the proportion of responders was 82.4% for both subclasses in the adjuvanted FLU-v group compared to 4.2% for IgG1 and 0% for IgG3 in the adjuvanted placebo group (*p <* 0.0001 for both), whereas the non-adjuvanted FLU-v group had 32.8% and 15.5% responders compared to 3.1% and 0% in the non-adjuvanted placebo group (*p =* 0.007 and 0.0151) (Table 2).

**Figure 1:**
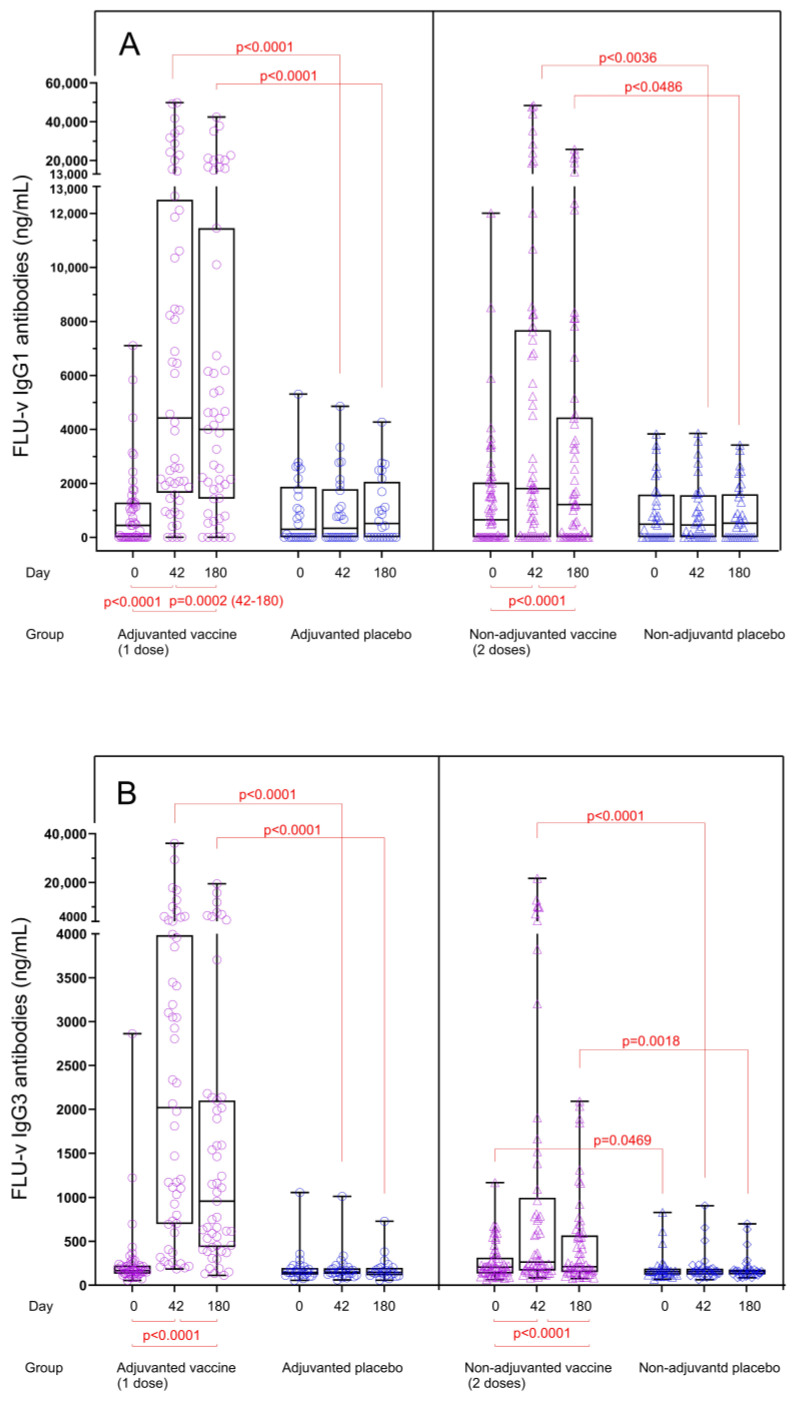
FLU-v IgG1 (**A**) and IgG3 (**B**) concentrations across all groups on days 0, 42, and 180. Data from the following groups are shown: adjuvanted FLU-v (*n* = 52), non-adjuvanted FLU-v (*n* = 58), adjuvanted placebo (*n* = 26), and non-adjuvanted placebo (*n* = 32). Box and whisker plot showing all individual values, 25th and 75th percentiles, and median. Whiskers represent ranges. The Mann–Whitney U test was used to compare different groups at each time point. The Wilcoxon signed-rank sum test was used to compare differences between time points within each group. Only significant differences are shown.

Quantitation of vaccine-induced IgG subclass levels: A single dose of adjuvanted FLU-v vaccine induced significant increases in both IgG1 and IgG3 concentrations after vaccination. From day 0 to day 42, IgG1 median concentration increased from 434 to 4422 ng/mL (*p <* 0.0001) (Figure 1A), whereas the concentration of IgG3 increased from 167 to 2020 ng/mL (*p <* 0.0001) (Figure 1B). At day 180, the median IgG1 concentration was sustained at 3999 ng/mL (*p <* 0.0001) (Figure 1A), whereas the median IgG3 concentration decreased to 958 ng/mL (*p <* 0.0001) (Figure 1B). No increase for any of the subclasses measured was seen in the adjuvanted placebo group.

Non-adjuvanted FLU-v vaccination also induced a significant but more modest increase in median concentrations of both IgG1 and IgG3 (Figure 1A,B). The median IgG1 level increased from 654 (day 0) to 1808 ng/mL on day 42 (*p <* 0.0001) and to 1219 ng/mL on day 180 (*p <* 0.0001). For IgG3 antibodies, the median levels increased from 206 (day 0) to 264 ng/mL on day 42 (*p <* 0.0001) and to 211 ng/mL on day 180 (*p <* 0.0001). Several individuals showed substantial pre-vaccination IgG1 levels (Figure 1A). No significant increases were detected in the non-adjuvanted placebo group after vaccination.

**Figure 2:**
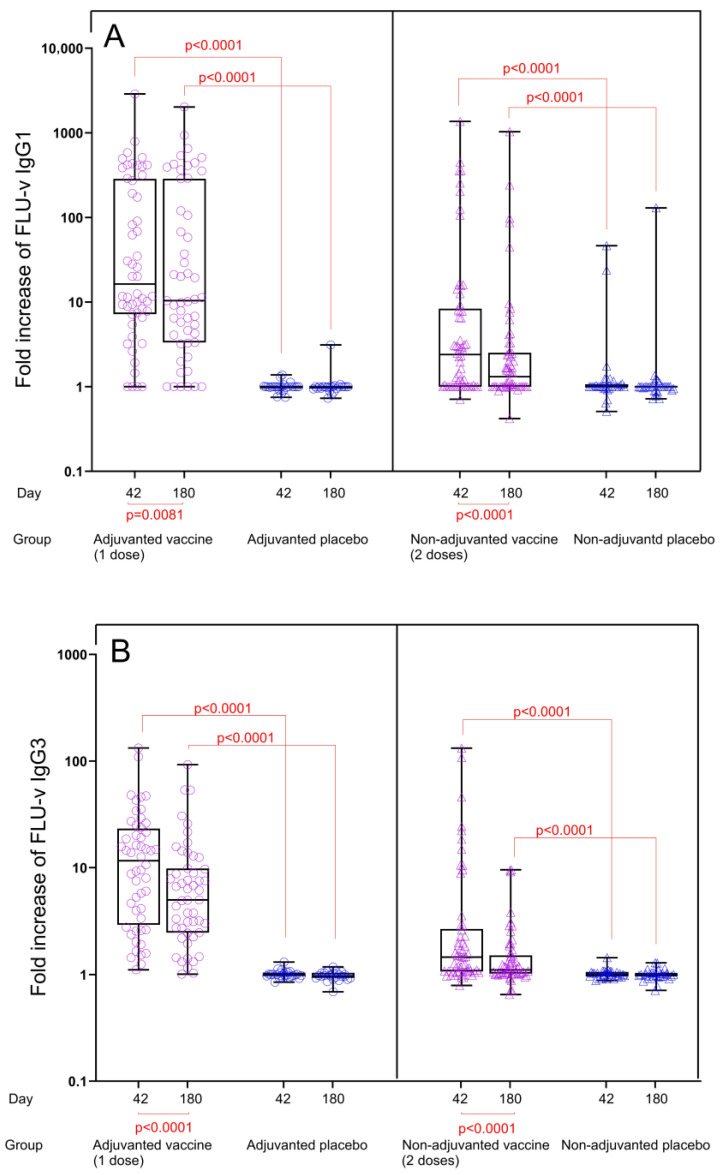
Fold increase in FLU-v IgG1 (**A**) and IgG3 (**B**) concentrations across all groups from day 0 to days 42 and 180. Data from the following groups are shown: adjuvanted FLU-v (*n* = 52), non-adjuvanted FLU-v (*n* = 58), adjuvanted placebo (n = 26), and non-adjuvanted placebo (*n* = 32). Box and whiskers plot showing all individual values, 25th and 75th percentiles, and median. Whiskers represent ranges. Fold change in IgG1 concentrations on day 42 and 180 is defined as the ratio of day 42 to day 0 and the ratio of day 180 to day 0, respectively. The Mann–Whitney U test was used to compare different groups at each time point. The Wilcoxon signed-rank sum test was used to compare differences between day 42 and 180 within each group. Significant differences are shown.

Fold changes in IgG subclass levels: IgG1 and IgG3 antibody levels in both vaccine groups were significantly increased compared to the corresponding placebo groups for both time points, and consequently, the fold changes for IgG1 and IgG3 concentrations from pre to post vaccination at both time points were also significantly higher in the vaccine groups compared to the placebo groups (*p <* 0.0001) (Figure 2A,B). In the adjuvanted FLU-v group, fold increases in IgG1 and IgG3 concentrations from day 0 to day 42 were 16.4- and 11.6-fold, whereas fold increases from day 0 to day 180 were 10.4- and 5.0-fold, respectively. Consistent with the results from quantitation, IgG1 and IgG3 fold increases in the non-adjuvanted group were considerably lower: 2.4- and 1.5-fold on day 42 and 1.3- and 1.1-fold on day 180, respectively. Although a reduction in IgG subclass concentrations in serum could be seen from day 42 to day 180 (IgG1: from 4422 to 3999 ng/mL, *p =* 0.0002 and IgG3: from 2020 to 958 ng/mL, *p <* 0.0001), substantial levels of both IgG subclasses were still present 6 months after adjuvanted vaccination (Figure 1A,B).

### 3.3. Adjuvanted and Non-Adjuvanted FLU-v Induce Antibody-Mediated NK Cell Activation

**Figure 3:**
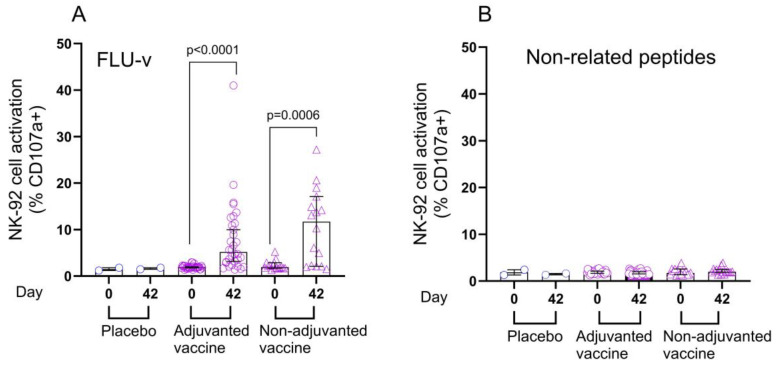
Antibody-mediated NK cell activation after adjuvanted and non-adjuvanted FLU-v vaccination. For this analysis, a subset of paired samples (*n* = 50) from day 0 and day 42 was selected based on high IgG1 and IgG3 fold increases. Data from the following groups are shown: adjuvanted FLU-v (*n* = 32), non-adjuvanted FLU-v (*n* = 16), and non-adjuvanted placebo (*n* = 2). Antibody-dependent NK cell activation, or percentage of GFP^+^ CD107a^+^ CD16 (V176) NK-92 cells by flow cytometry, was measured in response to antibodies immobilized by plate-bound FLU-v (**A**) or non-related peptides used as negative controls (**B**). Medians ± 95% confidences intervals are shown. The Wilcoxon signed-rank sum test was used to compare differences between time points within each vaccine group. A Bonferroni test was used to correct for multiple comparisons, with a *p* value < 0.025 considered significant.

To determine the ability of FLU-v-specific antibodies to induce NK cell activation, a subset of volunteers was selected based on the level of IgG1 and IgG3 fold increases on day 42 post vaccination. In this subset, NK-cell activation induced by FLU-v-specific antibodies present in serum collected pre vaccination was compared to the activation induced on day 42 post vaccination. Sera from participants that were vaccinated with adjuvanted FLU-v (*n* = 32) or non-adjuvanted FLU-v (*n* = 16) showed 2.7-fold and 6.2-fold median rises, respectively, in the percentage of NK cells expressing CD107a from day 0 to day 42 post vaccination. In the adjuvanted FLU-v group, median percentage of CD107a^+^ NK cells rose from 1.9% to 5.2% (*p <* 0.0001), whereas the non-adjuvanted group showed an increase from 1.9% to 11.7% (*p =* 0.0006) (Figure 3). No statistically significant difference in NK cell activation was found between the adjuvanted and non-adjuvanted vaccine groups. No rise in antibody-dependent NK cell activation was observed for placebo vaccinated (*n* = 2) or FLU-v vaccinated participants tested against non-related peptides used as negative controls (Figure 3).

### 3.4. FLU-v-Peptide-Specific Total IgG, IgG1 and IgG3 Correlate with Antibody-Mediated NK Cell Activation

**Figure 4:**
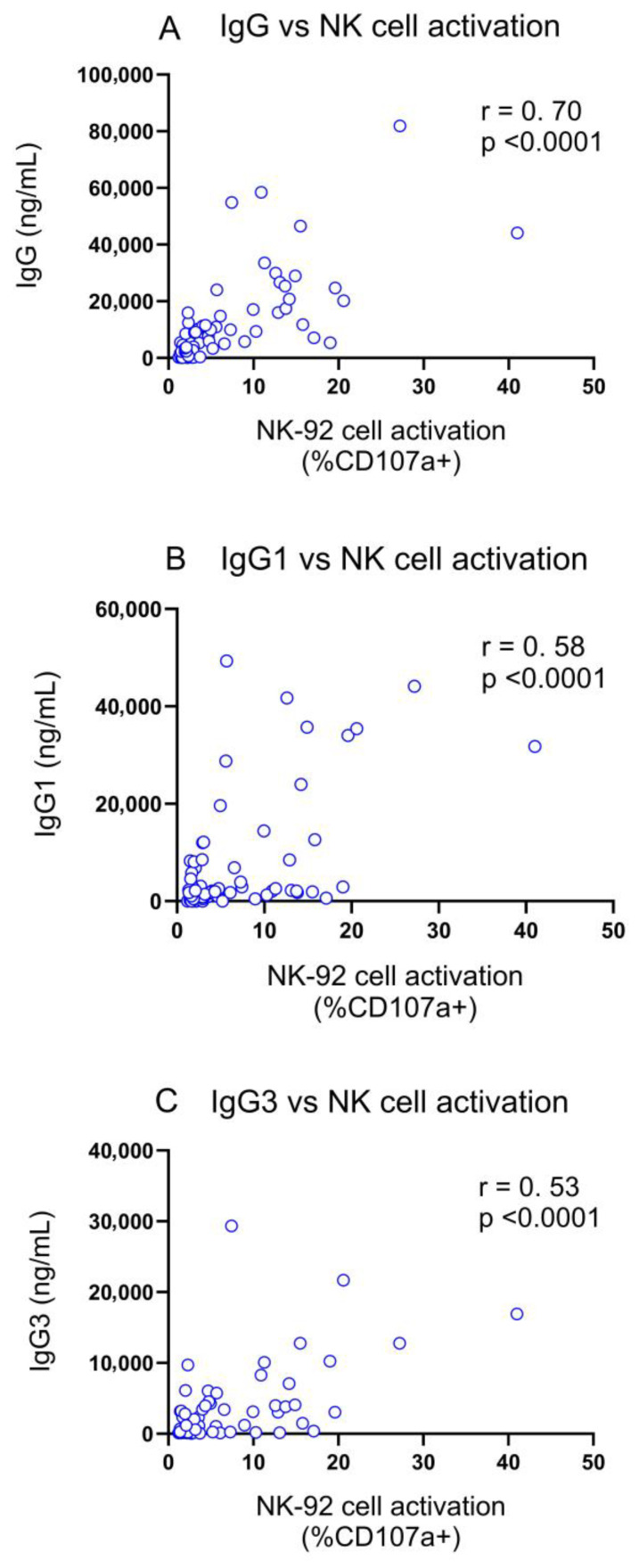
Correlation between antibody-mediated NK cell activation and FLU-v-specific IgG (**A**), IgG1 (**B**) and IgG3 (**C**) on days 0 and 42. Spearman rank order correlations were performed between the antibody-dependent NK cell activation assay, measuring the percentage of GFP^+^ CD107a^+^ CD16 (V176) NK-92 cells by flow cytometry, and total IgG, IgG1, and IgG3 with correlation coefficients (r) and *p*-values shown. IgG data for this analysis were obtained from a previous report [7].

Correlation analyses were carried out to determine any differences between the IgG1 and IgG3 subclasses with regard to the observed NK cell activation. Correlation analyses were performed between the concentration of FLU-v-specific IgG, IgG1, or IgG3 on day 42 and the percentage of CD107a^+^ NK-cells, which is an indicator of NK cell activation. The strongest correlation was found between FLU-v-specific total IgG and the percentage of CD107a^+^ NK cells (r = 0.70, *p <* 0.0001) (Figure 4A). Significant correlations were also observed between the percentage of CD107a^+^ NK cells and FLU-v-specific IgG1 and IgG3 concentrations (r = 0.58 (*p <* 0.0001) and r = 0.53 (*p <* 0.0001), respectively) (Figure 4 B,C).

## 4. Discussion

Although the FLU-v peptide vaccine was designed to induce T cell-mediated immunity, we have previously shown that both Montanide ISA-51-adjuvanted and -non-adjuvanted FLU-v induced significant vaccine-specific IgG responses measured by ELISA [7]. Here, we have further investigated the antibody response by quantifying IgG1 and IgG3 antibodies using an in-house-developed and validated vaccine-specific ELISA method. Since FLU-v only contains peptides from internal viral proteins (NP, M1, and M2), the IgG antibodies produced do not have neutralizing activity, but could exert protective effects by ADCC, complement-mediated responses, or opsonophagocytosis, which could lead to the destruction of cells infected by influenza virus [14]. In particular, the role of ADCC in protective immune responses against influenza has become evident in recent years, and this mechanism has consequently been an important target for vaccines aiming to induce antibodies with broad specificities, recognizing non-surface epitopes common to a variety of influenza strains [21,22]. Data from mouse models [23,24,25,26] and human trials demonstrate that NK cell activation and ADCC induced by cross-reactive antibodies directed against the conserved influenza antigens M2 and NP may play a role in protection against influenza infection and disease [17,27], thereby providing an attractive approach for developing universal influenza vaccines based on antibody-mediated cross-protection, in addition to T cell immunity [21].

The aim of this study was to dissect the composition of the antibody response induced by FLU-v vaccination by focusing on the IgG1 and IgG3 subclasses, which both induce ADCC responses via NK cell activation [13]. We have demonstrated that both adjuvanted and non-adjuvanted FLU-v vaccination induced robust IgG1 and IgG3 responses. Although a direct comparison between the two vaccine groups is theoretically compromised by a different number of vaccine doses given (one dose of adjuvanted vs. two doses of non-adjuvanted vaccine), the results nevertheless suggest an enhancing effect of the adjuvant used here for both IgG subclass responses. Enhancement of IgG antibody responses has also been shown when the same adjuvant, Montanide ISA-51, has been used in other vaccines [28], including a licensed therapeutic lung cancer vaccine [29]. The quantitative distribution of IgG1 and IgG3 responders in both vaccine groups showed a large degree of variation, ranging from low to high responders, which is often seen in influenza vaccination of individuals with varying degree of pre-existing antibodies due to natural exposure [30]. Consistent with the hypothesis that the majority of the adult population has been naturally exposed to influenza viruses, we observed that some individuals had pre-existing IgG1 antibodies to the vaccine antigens. This observation indicates that processing the whole virus during natural infection can result in successful presentation of the antigen epitopes recognized by FLU-v-induced antibodies, thereby supporting the prospects for functional antibody-mediated protection against influenza disease induced by this vaccine.

Importantly, in the adjuvanted FLU-v group, both IgG1 and IgG3 levels persisted over time, with only moderate reductions in antibody levels in more than 80% of the vaccinees at 6 months compared to day 42 post vaccination. However, the decline was more pronounced for IgG3 than for IgG1, which is expected due to the shorter half-life of IgG3 caused by reduced affinity for the neonatal Fc receptor and increased susceptibility to proteolytic cleavage. This is a known mechanism limiting the potent pro-inflammatory effects of IgG3 antibodies [31]. Long-term protection is pivotal for a broad-spectrum influenza vaccine, and the duration beyond six months of FLU-v-specific IgG1 and IgG3 antibodies will have to be further evaluated in future studies. Moreover, it would also be important to determine whether exposure to influenza virus post vaccination is capable of boosting FLU-v-specific antibody levels once memory B cells have been generated by FLU-v vaccination.

Demonstrating that FLU-v induces IgG1 and IgG3 antibodies does not necessarily mean that these antibodies play a role in protection against influenza infection. For this reason, their ability to induce FcγRIIIa-mediated NK cell activation *in vitro* was evaluated and shown to increase from pre to post vaccination in both vaccine groups. NK cell activating antibodies have recently been presented as a correlate of protective immunity against influenza infection [19], and the relevance of this assay for evaluating ADCC capacity of vaccine-induced antibodies is well established [18,21]. Cross-linking FcγRIIIa on human NK cells leads to NK cell activation, which has been shown to correlate with an increase in ADCC responses, resulting in the killing of host cells infected by the virus [32,33]. Moreover, it is important to note that testing sera from virus-exposed and naturally pre-immunized vaccinated subjects in any ADCC assay with influenza-infected target cells would not be methodologically appropriate, since infected cells also express other ADCC-inducing antigens (like HA and NA), masking the NP- and M- specific ADCC activity we wanted to detect for FLU-v antibodies.

Although we do not know which of the antigen targets in the vaccine (NP, M1, or M2) that contributes to the observed NK cell activation, human anti-NP IgG antibodies induced by natural or experimental infection have been shown to activate NK cells by FcγRIIIa engagement [18]. Several studies in mice have also shown that antibodies against influenza proteins M2 and NP contribute to heterosubtypic immunity and protection against virus challenge by activating an FcγR-dependent mechanism [24,25,27]. Less is known in humans about the role of the M1 and M2 proteins in this context, but human anti-M2 monoclonal antibodies with proven therapeutic potential have been demonstrated to activate NK cells and mediate ADCC [17,26]. Based on the knowledge that IgG3 is more efficient than IgG1 in mediating effector functions [34], the substantial IgG3 response induced by adjuvanted FLU-v vaccination may be relevant for protection. In particular, IgG3 antibodies have been found to bind FcγRIIIa with approximately three times higher affinity than IgG1 [35], implying that IgG3 antibodies can activate FcγRIIIa-expressing NK cells more efficiently than the IgG1 subclass.

A limitation of this study is that only a subset of samples was used for evaluating NK cell activation by selecting participants with low pre-existing antibodies and high post-vaccination FLU-v antibody levels, thereby primarily focusing on the ability of the vaccine-induced antibodies to induce NK cell activation. This subset selection may have influenced the results from comparing NK cell activation in the adjuvanted and non-adjuvanted vaccine group. Moreover, we were not able to elucidate any potential difference in the relative ability of the IgG1 and IgG3 subclasses to induce NK cell activation due to the presence of both FLU-v-specific IgG1 and IgG3 antibodies in most sera tested. Further studies are needed to evaluate the relative importance of FLU-v-specific IgG3 versus IgG1 in NK cell activation, as well as a potential difference in the glycosylation pattern and epitope specificity between the two subclasses. Separation of the subclasses and coating with individual peptides rather than using the peptide mix may provide more information on the individual epitopes and IgG subclasses involved in this effector function. Moreover, it will be important to investigate whether FLU-v-specific antibodies isolated from serum can induce cytotoxicity against influenza-infected host cells *in vitro* in functional ADCC assays. By using different seasonal and pandemic influenza strains to infect target cells, such assays could also provide crucial information on the broadness of ADCC-mediated immunity induced by this vaccine.

Although T cell-mediated immunity has been an important target for the development of broad-spectrum influenza vaccines utilizing conserved antigens [36], there is also a need for exploration of potential protective antibody responses to broaden the repertoire of effector functions. Since most neutralizing antibodies directed against HA are highly strain-specific, vaccine candidates addressing the protective potential of ADCC and other Fc-mediated effector functions may be a fruitful approach for including antibody-based immunity in broad-spectrum influenza vaccines. The added value of this component is the elimination of virus-infected host cells, thereby providing protection against disease severity. While many efforts are focusing on HA stem-directed cross-reactive antibody responses, with both membrane fusion blocking and FcR-mediated effector functions as promising targets for protection [37,38,39], the more conserved M1, M2, and NP antigens have also successfully been used in clinical trials with different vaccine platforms aiming to induce both T cell- and antibody-mediated immunity [7,36,39,40]. Although FLU-v was primarily designed to induce T cell-mediated immunity, this vaccine is, to our knowledge, the first peptide-based broad-spectrum influenza vaccine candidate to demonstrate induction of NK cell-activating IgG antibodies, thereby broadening the immunological mechanisms contributing to protection against influenza for this vaccine platform.

## 5. Conclusions

An in-house-developed and -validated ELISA method was used for quantitation of FLU-v peptide-specific IgG1 and IgG3 subclass antibodies in human serum after FLU-v vaccination. Montanide ISA-51-adjuvanted FLU-v vaccination induced durable IgG1 and IgG3 antibodies after a single dose in almost 90% of the vaccinees. The demonstrated ability of FLU-v-induced antibodies to activate NK cells suggests a potential role in ADCC-mediated protection against influenza after FLU-v peptide vaccination.

## Figures and Tables

**Table 1:** Amino acid sequences of the peptides included in the FLU-v vaccine and the protein they originate from.

Peptide Name	Protein Origin	Amino Acid Sequence
FLU-5 acetate	M1 protein	DLEALMEWLKTRPILSPLTKGILGFVFTLTVP
FLU-7 acetate	NP protein from influenza A strains	DLIFLARSALILRGSVAHKS
FLU-8N acetate	NP protein from influenza B strains	PGIADIEDLTLLARSMVVVR
FLU-10 acetate	M2 protein	IIGILHLILWILDRLFFKCIYRLF

**Table 2:** Number and percentage of vaccine responders for FLU-v-vaccine-specific IgG1 and IgG3 subclass antibodies. Vaccine responders (*r*) are defined as participants reaching a two-fold or higher increase in IgG1/IgG3 concentration (ng/mL) from day 0 to day 42 or from day 0 to day 180. *n* = number of participants with serum sample analyzed. Percentage responders were calculated with corresponding 95% Wilson score confidence intervals (CIs).

Number of IgG1/IgG3 Responders	Adjuvanted FLU-v (1 Dose) vs. Adjuvanted Placebo			Non-Adjuvanted FLU-v (2 Doses) vs. Non-Adjuvanted Placebo		
	Adjuvanted FLU-v	Adjuvanted Placebo	*p*-Value Fisher Mid-P Test	Non-Adjuvanted FLU-v	Non- Adjuvanted Placebo	*p*-Value Fisher Mid-P Test
**IgG1 Day 42**						
Proportion, *r/n*	46/52	0/26	**<0.0001**	31/58	2/32	**<0.0001**
(**% responders)**	(**88.5%)**	**(0.0%)**		(**53.4%)**	**(6.3%)**	
(95% CI)	(77.0–94.6)	(0.0–12.9)		(40.8–65.7)	(1.7–20.1)	
**IgG1 Day 180**						
Proportion, *r/n*	42/51	1/24	**<0.0001**	19/58	1/32	**0.0007**
**(% responders)**	**(82.4%)**	**(4.2%)**		**(32.8%)**	(**3.1%)**	
(95% CI)	(69.7–90.4)	(0.2–20.2)		(22.1–45.6)	(0.2–15.7)	
**IgG3 Day 42**						
Proportion, *r/n*	45/52	0/26	**<0.0001**	7/58	0/32	**0.0002**
**% responders**	**(86.5%)**	**(0.0%)**		**(29.3%)**	(**0.0%)**	
(95% CI)	(74.7–93.3)	(0.0–12.9)		(19.2–42.0)	(0.0–10.7)	
**IgG3 Day 180**						
Proportion, *r/n*	42/51	0/24	**<0.0001**	9/58	0/32	**0.0151**
**% responders**	(**82.4%)**	**(0.0%)**		**(15.5%)**	**(0.0%)**	
(95% CI)	(69.7–90.4)	(0.0–13.8)		(8.4–26.9)	(0.0–10.7)	

## Data Availability

Archives of the data collected are not publicly available.

## Supplementary Materials

The following supporting information can be downloaded at: https://www.mdpi.com/article/10.3390/vaccines13111084/s1, Table S1: Validation of IgG subclass ELISA: intra-assay precision for IgG1 and IgG3. Table S2: Validation of IgG subclass ELISA: inter-assay (intermediate precision) for IgG1. Table S3: Validation of IgG subclass ELISA: inter-assay (intermediate precision for IgG3).

## References

[1] Paules C.I., Sullivan S.G., Subbarao K., Fauci A.S. (2018). Chasing Seasonal Influenza—The Need for a Universal Influenza Vaccine. N. Engl. J. Med..

[2] van Els C., Mjaaland S., Næss L., Sarkadi J., Gonczol E., Korsholm K.S., Hansen J., de Jonge J., Kersten G., Warner J. (2014). Fast vaccine design and development based on correlates of protection (COPs). Hum. Vaccin. Immunother..

[3] Stoloff G.A., Caparros-Wanderley W. (2007). Synthetic multi-epitope peptides identified in silico induce protective immunity against multiple influenza serotypes. Eur. J. Immunol..

[4] Behrendt R., White P., Offer J. (2016). Advances in Fmoc solid-phase peptide synthesis. J. Pept. Sci..

[5] Pleguezuelos O., Robinson S., Stoloff G.A., Caparrós-Wanderley W. (2012). Synthetic Influenza vaccine (FLU-v) stimulates cell mediated immunity in a double-blind, randomised, placebo-controlled Phase I trial. Vaccine.

[6] van Doorn E., Pleguezuelos O., Liu H., Fernandez A., Bannister R., Stoloff G., Oftung F., Norley S., Huckriede A., Frijlink H.W. (2017). Evaluation of the immunogenicity and safety of different doses and formulations of a broad spectrum influenza vaccine (FLU-v) developed by SEEK: Study protocol for a single-center, randomized, double-blind and placebo-controlled clinical phase IIb trial. BMC Infect. Dis..

[7] Pleguezuelos O., Dille J., de Groen S., Oftung F., Niesters H.G.M., Islam M.A., Næss L.M., Hungnes O., Aldarij N., Idema D.L. (2020). Immunogenicity, Safety, and Efficacy of a Standalone Universal Influenza Vaccine, FLU-v, in Healthy Adults: A Randomized Clinical Trial. Ann. Intern. Med..

[8] Oftung F., Næss L.M., Laake I., Stoloff G., Pleguezuelos O. (2022). FLU-v, a Broad-Spectrum Influenza Vaccine, Induces Cross-Reactive Cellular Immune Responses in Humans Measured by Dual IFN-γ and Granzyme B ELISpot Assay. Vaccines.

[9] Pleguezuelos O., James E., Fernandez A., Lopes V., Rosas L.A., Cervantes-Medina A., Cleath J., Edwards K., Neitzey D., Gu W. (2020). Efficacy of FLU-v, a broad-spectrum influenza vaccine, in a randomized phase IIb human influenza challenge study. NPJ Vaccines.

[10] Aucouturier J., Dupuis L., Deville S., Ascarateil S., Ganne V. (2002). Montanide ISA 720 and 51: A new generation of water in oil emulsions as adjuvants for human vaccines. Expert Rev. Vaccines.

[11] Liu H., Frijlink H.W., Huckriede A., van Doorn E., Schmidt E., Leroy O., Rimmelzwaan G., McCullough K., Whelan M., Hak E. (2016). Influenza Vaccine Research funded by the European Commission FP7-Health-2013-Innovation-1 project. Vaccine.

[12] Krammer F. (2019). The human antibody response to influenza A virus infection and vaccination. Nat. Rev. Immunol..

[13] Ferrante A., Beard L.J., Feldman R.G. (1990). IgG subclass distribution of antibodies to bacterial and viral antigens. Pediatr. Infect. Dis. J..

[14] Vidarsson G., Dekkers G., Rispens T. (2014). IgG subclasses and allotypes: From structure to effector functions. Front. Immunol..

[15] Boudreau C.M., Alter G. (2019). Extra-Neutralizing FcR-Mediated Antibody Functions for a Universal Influenza Vaccine. Front. Immunol..

[16] Von Holle T.A., Moody M.A. (2019). Influenza and Antibody-Dependent Cellular Cytotoxicity. Front. Immunol..

[17] Simhadri V.R., Dimitrova M., Mariano J.L., Zenarruzabeitia O., Zhong W., Ozawa T., Muraguchi A., Kishi H., Eichelberger M.C., Borrego F. (2015). A Human Anti-M2 Antibody Mediates Antibody-Dependent Cell-Mediated Cytotoxicity (ADCC) and Cytokine Secretion by Resting and Cytokine-Preactivated Natural Killer (NK) Cells. PLoS ONE.

[18] Vanderven H.A., Ana-Sosa-Batiz F., Jegaskanda S., Rockman S., Laurie K., Barr I., Chen W., Wines B., Hogarth P.M., Lambe T. (2016). What Lies Beneath: Antibody Dependent Natural Killer Cell Activation by Antibodies to Internal Influenza Virus Proteins. EBioMedicine.

[19] Boudreau C.M., Burke J.S.T., Yousif A.S., Sangesland M., Jastrzebski S., Verschoor C., Kuchel G., Lingwood D., Kleanthous H., De Bruijn I. (2023). Antibody-mediated NK cell activation as a correlate of immunity against influenza infection. Nat. Commun..

[20] Gong J.H., Maki G., Klingemann H.G. (1994). Characterization of a human cell line (NK-92) with phenotypical and functional characteristics of activated natural killer cells. Leukemia.

[21] Jegaskanda S. (2018). The Potential Role of Fc-Receptor Functions in the Development of a Universal Influenza Vaccine. Vaccines.

[22] Gao R., Sheng Z., Sreenivasan C.C., Wang D., Li F. (2020). Influenza A Virus Antibodies with Antibody-Dependent Cellular Cytotoxicity Function. Viruses.

[23] DiLillo D.J., Tan G.S., Palese P., Ravetch J.V. (2014). Broadly neutralizing hemagglutinin stalk-specific antibodies require FcgammaR interactions for protection against influenza virus in vivo. Nat. Med..

[24] Carragher D.M., Kaminski D.A., Moquin A., Hartson L., Randall T.D. (2008). A novel role for non-neutralizing antibodies against nucleoprotein in facilitating resistance to influenza virus. J. Immunol..

[25] LaMere M.W., Lam H.T., Moquin A., Haynes L., Lund F.E., Randall T.D., Kaminski D.A. (2011). Contributions of antinucleoprotein IgG to heterosubtypic immunity against influenza virus. J. Immunol..

[26] Wang R., Song A., Levin J., Dennis D., Zhang N.J., Yoshida H., Koriazova L., Madura L., Shapiro L., Matsumoto A. (2008). Therapeutic potential of a fully human monoclonal antibody against influenza A virus M2 protein. Antiviral. Res..

[27] El Bakkouri K., Descamps F., De Filette M., Smet A., Festjens E., Birkett A., Van Rooijen N., Verbeek S., Fiers W., Saelens X. (2011). Universal vaccine based on ectodomain of matrix protein 2 of influenza A: Fc receptors and alveolar macrophages mediate protection. J. Immunol..

[28] Yamshchikov G.V., Barnd D.L., Eastham S., Galavotti H., Patterson J.W., Deacon D.H., Teates D., Neese P., Grosh W.W., Petroni G. (2001). Evaluation of peptide vaccine immunogenicity in draining lymph nodes and peripheral blood of melanoma patients. Int. J. Cancer.

[29] Saavedra D., Crombet T. (2017). CIMAvax-EGF: A New Therapeutic Vaccine for Advanced Non-Small Cell Lung Cancer Patients. Front. Immunol..

[30] Hinojosa M., Shepard S.S., Chung J.R., King J.P., McLean H.Q., Flannery B., Belongia E.A., Levine M.Z. (2021). Impact of Immune Priming, Vaccination, and Infection on Influenza A(H3N2) Antibody Landscapes in Children. J. Infect. Dis..

[31] Chu T.H., Patz E.F., Ackerman M.E. (2021). Coming together at the hinges: Therapeutic prospects of IgG3. MAbs.

[32] Vanderven H.A., Liu L., Ana-Sosa-Batiz F., Nguyen T.H., Wan Y., Wines B., Hogarth P.M., Tilmanis D., Reynaldi A., Parsons M.S. (2017). Fc functional antibodies in humans with severe H7N9 and seasonal influenza. JCI Insight.

[33] Vanderven H.A., Wragg K., Ana-Sosa-Batiz F., Kristensen A.B., Jegaskanda S., Wheatley A.K., Wentworth D., Wines B.D., Hogarth P.M., Rockman S. (2018). Anti-Influenza Hyperimmune Immunoglobulin Enhances Fc-Functional Antibody Immunity During Human Influenza Infection. J. Infect. Dis..

[34] Damelang T., Rogerson S.J., Kent S.J., Chung A.W. (2019). Role of IgG3 in Infectious Diseases. Trends Immunol..

[35] Bruhns P., Iannascoli B., England P., Mancardi D.A., Fernandez N., Jorieux S., Daeron M. (2009). Specificity and affinity of human Fcgamma receptors and their polymorphic variants for human IgG subclasses. Blood.

[36] Corder B.N., Bullard B.L., Poland G.A., Weaver E.A. (2020). A Decade in Review: A Systematic Review of Universal Influenza Vaccines in Clinical Trials during the 2010 Decade. Viruses.

[37] Nachbagauer R., Feser J., Naficy A., Bernstein D.I., Guptill J., Walter E.B., Berlanda-Scorza F., Stadlbauer D., Wilson P.C., Aydillo T. (2021). A chimeric hemagglutinin-based universal influenza virus vaccine approach induces broad and long-lasting immunity in a randomized, placebo-controlled phase I trial. Nat. Med..

[38] Nachbagauer R., Palese P. (2018). Development of next generation hemagglutinin-based broadly protective influenza virus vaccines. Curr. Opin. Immunol..

[39] Yamayoshi S., Kawaoka Y. (2019). Current and future influenza vaccines. Nat. Med..

[40] Leroux-Roels I., Waerlop G., Tourneur J., De Boever F., Maes C., Bruhwyler J., Guyon-Gellin D., Moris P., Del Campo J., Willems P. (2022). Randomized, Double-Blind, Reference-Controlled, Phase 2a Study Evaluating the Immunogenicity and Safety of OVX836, A Nucleoprotein-Based Influenza Vaccine. Front. Immunol..

