# Supplementary material for: FLU-v, a Broad-Spectrum Peptide-Based Influenza Vaccine, Induces NK Cell Activating IgG1 and IgG3 Subclass Antibodies in Humans
Source: Vaccines (Basel). 2025 Oct 22;13(11):1084. doi: 10.3390/vaccines13111084 (PMC12656455; doi:10.3390/vaccines13111084)
Supplement: Supplementary file 1 [file vaccines-13-01084-s001.zip › vaccines-3904016-supplementary.pdf]

SUPPLEMENTARY

Validation of ELISA – Precision results

Table S1. Intra-assay precision to measure repeatability expressed as % CV (coefficient of variation).

IgG1 ref	1000 ng/mL	500	250	125	62.50	31.25	15.63	7.81	3.91	1.95	BLANK
**Run 1**	2.80	3.55	1.67	2.92	5.78	1.72	1.64	2.69	1.86	1.93	3.30
**Run 2**	2.53	5.21	5.92	2.79	3.82	2.52	5.78	1.49	2.91	3.54	8.23
**Run 3**	2.23	3.13	1.83	2.68	3.89	7.97	3.63	4.08	1.71	8.10	2.44
**IgG3 ref**	**1000 ng/mL**	**500**	**250**	**125**	**62.50**	**31.25**	**15.63**	**7.81**	**3.91**	**1.95**	**BLANK**
**Run 1**	1.77	1.74	2.68	1.34	2.70	2.69	3.48	2.53	2.06	1.60	1.22
**Run 2**	1.82	2.13	2.22	1.17	2.26	4.43	3.29	2.78	1.40	1.36	4.28
**Run 3**	1.26	2.30	4.24	2.67	3.64	4.09	4.15	2.57	2.24	1.94	2.44

CVs are based on 8 replicates on the same ELISA plate for IgG1 and IgG3 references and blank wells

Table S2. Inter-assay (intermediate) precision expressed as mean OD values, SD and % CV for IgG1 reference.

IgG1 ref	1000 ng/mL	500	250	125	62.50	31.25	15.63	7.81	3.91	1.95
**Run 1**	2.464	1.347	0.639	0.288	0.145	0.088	0.065	0.055	0.049	0.047
**Run 2**	2.657	1.323	0.727	0.332	0.157	0.099	0.070	0.056	0.050	0.048
**Run 3**	2.585	1.440	0.653	0.254	0.127	0.084	0.064	0.054	0.049	0.049
**Mean**	2.569	1.370	0.673	0.291	0.143	0.090	0.066	0.055	0.049	0.048
**SD**	0.098	0.062	0.047	0.039	0.015	0.008	0.003	0.001	0.001	0.001
**CV(%)**	3.80	4.51	7.03	13.42	10.56	8.60	4.85	1.82	1.17	2.08

Table S3. Inter-assay (intermediate) precision expressed as mean OD values, SD and % CV for IgG3 reference.

IgG3 ref	1000 ng/mL	500	250	125	62.50	31.25	15.63	7.81	3.91	1.95
**Run 1**	2.035	1.720	1.260	0.925	0.652	0.428	0.261	0.156	0.102	0.074
**Run 2**	1.915	1.605	1.240	0.898	0.625	0.415	0.249	0.154	0.101	0.073
**Run 3**	1.729	1.426	1.082	0.797	0.557	0.368	0.229	0.142	0.095	0.072
**Mean**	1.893	1.584	1.194	0.873	0.611	0.404	0.246	0.151	0.099	0.073
**SD**	0.154	0.148	0.098	0.067	0.049	0.032	0.016	0.008	0.004	0.001
**CV(%)**	8.14	9.36	8.17	7.73	8.01	7.82	6.56	5.03	3.81	1.37
